# Intelligent System for Detecting Deterioration of Life Satisfaction as Tool for Remote Mental-Health Monitoring

**DOI:** 10.3390/s22239214

**Published:** 2022-11-26

**Authors:** Piotr Prokopowicz, Dariusz Mikołajewski, Emilia Mikołajewska

**Affiliations:** 1 Institute of Computer Science, Kazimierz Wielki University, 85-064 Bydgoszcz, Poland; 2 Laboratory of Neurophysiological Research, Medical University of Lublin, 20-059 Lublin, Poland; 3 Faculty of Health Sciences, Ludwik Rydygier Collegium Medicum in Bydgoszcz, Nicolaus Copernicus University in Toruń, 87-100 Toruń, Poland

**Keywords:** intelligent systems, computational model, fuzzy logic, remote monitoring, quality of life, life satisfaction, burnout, COVID-19, predictive analytics

## Abstract

The research described in this article is a continuation of work on a computational model of quality of life (QoL) satisfaction. In the proposed approach, overall life satisfaction is aggregated to personal life satisfaction (PLUS). The model described in the article is based on well-known and commonly used clinimetric scales (e.g., in psychiatry, psychology and physiotherapy). The simultaneous use of multiple scales, and the complexity of describing the quality of life with them, require complex fuzzy computational solutions. The aim of the study is twofold: (1) To develop a fuzzy model that allows for the detection of changes in life satisfaction scores (data on the influence of the COVID-19 pandemic and the war in the neighboring country were used). (2) To develop more detailed guidelines than the existing ones for further similar research on more advanced intelligent systems with computational models which allow for sensing, detecting and evaluating the psychical state. We are concerned with developing practical solutions with higher scientific and clinical utility for both small datasets and big data to use in remote patient monitoring. Two exemplary groups of specialists at risk of occupational burnout were assessed three times at different intervals in terms of life satisfaction. The aforementioned assessment was made on Polish citizens because the specific data could be gathered: before and during the pandemic and during the war in Ukraine (a neighboring country). That has a higher potential for presenting a better analysis and reflection on the practical application of the model. A research group (physiotherapists, n = 20) and a reference group (IT professionals, n = 20) participated in the study. Four clinimetric scales were used for assessment: the Perceived Stress Scale (PSS10), the Maslach Burnout Scale (MBI), the Satisfaction with Life Scale (SWLS), and the Nordic Musculoskeletal Questionnaire (NMQ). The assessment was complemented by statistical analyses and fuzzy models based on a hierarchical fuzzy system. Although several models for understanding changes in life satisfaction scores have been previously investigated, the novelty of this study lies in the use of data from three consecutive time points for the same individuals and the way they are analyzed, based on fuzzy logic. In addition, the new hierarchical structure of the model used in the study provides flexibility and transparency in the process of remotely monitoring changes in people’s mental well-being and a quick response to observed changes. The aforementioned computational approach was used for the first time.

## 1. Introduction

Recent times have been fraught with global events traumatizing and testing mental toughness. Pandemics, wars or economic crises and rising inflation affect life satisfaction, making it even more difficult to cope with the challenges of family life, work and community activities. This is compounded by concerns about the future; related to health or environmental quality.

Currently, the disparity between mental and physical health is not in doubt, leading to many problems within a biopsychosocial approach that treats the whole person rather than the illness. This is influenced by cultural attitudes, the risk of stigma in the community, discrimination in accessing mental health care, or even the structure of the health care system itself. The aforementioned imbalance between the treatment of mental disorders and the treatment of physical disorders causes inequalities in diagnosis, treatment and treatment outcomes. Statistical data can therefore be misleading here, causing further exacerbation of existing problems [1].

Developments in technology to support medical science, including artificial intelligence, are opening up new possibilities for mental health support and data collection. This may present an opportunity to reach new patients more widely, including those who have previously dropped out of diagnosis or discontinued therapy. Researchers and clinicians continue to search for technologies that are attractive to patients, more effective and that enable early detection and treatment of potentially harmful mental health changes [1]. This article is moving in that direction.

Remote patient monitoring offers to monitor the changes in citizens’ mental states and provide a rapid response to observed changes.

The main purpose of the present study is the detection of deterioration in life satisfaction. It can provide important information for the early detection of different mental problems, such as depressive states and behaviors or job burnout.

According to the authors’ review of the five largest bibliometric databases, the problem of defining quality of life has already been devoted to 10,769 papers published between 1968 and 2022. Similarly, between 1992 and 2022, 2972 publications were published on measuring the quality of life. Despite the wealth of knowledge on the subject, the dynamics of current events and the increasing uncertainty of citizens make it increasingly difficult to measure quality of life, and current computational models are incomplete or inaccurate. Artificial intelligence has the potential to provide new solutions for analyzing, modeling and predicting quality of life, but only 101 significant papers have been produced to date, including eighteen reviews, five clinical trials and no meta-analyses. The majority of these were in oncology, surgery and orthopedics, diabetology, and dietetics, and only three were concerned with the quality of life in psychiatric and neurodegenerative problems [2,3,4]. Work by Komatsu et al. has shown that machine learning (ML)-based approaches and multidimensional datasets allow for computational predefinition of mental illness, not only in the area of diagnosis but also in clinical explanation, therapy and prognosis [2]. In neurodegenerative conditions, research to date using artificial intelligence (AI) has focused on three objectives: testing the utility of AI in improving quality of life in ’Alzheimer’s disease, accuracy in detecting events (e.g., falls), and improving understanding of user needs regarding the functionality of future AI technologies [3].Moreover, in Alzheimer’s disease research, quality of life measures are often used as proxy indicators that do not take the individual preferences of patients into account, which may influence treatment outcomes [4]. Thanks to AI, clinicians and scientists can use complex patterns of research results and behavior to combine the abovementioned computational techniques, and data sets from repositories deepen the biological characteristics of health and its main disorders, including mental disorders. In evidence-based personalized psychiatry, tailored for individual patients, objective analysis, classification and prediction of phenotypes can allow for earlier diagnosis, individual treatment selection, and less disease impact [5]. Precision psychiatry, based on ML, provides patients with the right drugs at the right dose at the right time. Various ML approaches are used to predict diagnosis, prognosis and treatment in different cases, biomarkers involved in mental illness, and patient response to treatment [6]. Many diseases need to be identified as early as possible in order to start appropriate treatment and increase the chances of therapeutic success. Therefore, there is a need for automated analysis of medical data, including preventative medicine, i.e., the medicine of apparently healthy people [7].

Currently, the biggest challenge for computational techniques is to increase the accuracy of the algorithm while reducing the time required for its execution [7]; hence the constant search for more effective methods, techniques and computational algorithms adapted to biomedical data. Better patient care and objective resource allocation are guided using ML-based decision-making [7].

Although several models for understanding changes in life satisfaction scores have been previously investigated, the novelty of this study lies in the use of data from three consecutive time points for the same individuals and the way they are analyzed, based on fuzzy logic. In addition, the new hierarchical structure of the model used in the study provides flexibility and transparency for remotely monitoring changes in people’s mental well-being and responding quickly to observed changes. The aforementioned computational approach was used for the first time. Thus, our study fills the research and data analysis gap based on the results of the authors’ own research.

The computational model improves and speeds up the analysis of the data and allows for much broader inference and prediction from the data. Assessment of the quality of life or satisfaction with life is usually subjective and multifactorial (related, for example, to economic criteria [8,9]). For the aforementioned reasons, in the search for representative coefficients to support the detection of deterioration of life satisfaction and assessment of life satisfaction, we turned to clinimetric tests, taking into account both psychological aspects of life (e.g., job burnout) or the physical condition of the human body [10,11,12,13,14].Focusing on objectifying the individual’s sense of life satisfaction, a previous work introduced the ’personal living usual satisfaction’(PLUS) model [15]. Even though it is based on previously known and widely used solutions (clinimetric tests from psychology and physiotherapy), thanks to the use of fuzzy logic, it allows us to obtain a synergistic effect (aggregation) of knowledge from already existing procedures and a directional effect of changes in the values of the parameters tested and calculated. Data sources in the form of tests provide a linguistic description of the relationships and results using digital models based on fuzzy sets and fuzzy systems [16,17,18], defining rules based on the linguistic description. Assumptions and rules of the data sources that are impossible to describe with traditional mathematical models are preserved. So far, the hierarchical fuzzy systems model has proved effective in biomedical and industrial applications, which confirms the advisability of its selection for use in the present study [19,20,21,22,23]. The hierarchical modular design improves the deterioration detection and assessment procedures’ transparency, flexibility and scalability. The study relied on three different contexts of personal life: well-being and satisfaction, job satisfaction and performance, and physical well-being in activities of daily living (ADLs).Four tests were used in the study: Perceived Stress Score (PSS10), Maslach Burnout Inventory (MBI), Satisfaction with Life Scale (SWLS),and Nordic Musculoskeletal Questionnaire (NMQ). These are complementary to each other. The PSS10 is used to assess situations in a person’s life self-rated as stressful [24]. The MBI is used to measure job burnout within three areas (dimensions): emotional exhaustion (EE), depersonalization (DP) and personal achievement (PA) [25]. The SWLS is a tool for measuring general cognitive assessments of life satisfaction [26]. The NMQ- allows the assessment of musculoskeletal disorders, especially shoulder, neck, and lower back pain [27]. The aforementioned tests, frequently used in clinical practice, are valid and reliable [28,29,30,31], which favors the replication of our study.

Areas of particular interest in this study are the impact of the COVID-19 pandemic on Poles’ well-being and life satisfaction (including differences in possible job burnout and work-related stress) and war in a neighboring country. Both the COVID-19 pandemic [32,33,34,35,36] and the war in Ukraine [37,38] have put additional strain on the workforce, especially medical professionals, but the specific impact on the aforementioned workers remain unknown, despite research to date [34,35,36]. Furthermore, currently, we can mainly observe short-term effects, such as challenges to the care system in Poland [37,38,39,40,41] or the devastation of human rights [42]. To date, none of the studies combines clinical and computational approaches to the effects of the war in Ukraine on the health and quality of life of Poles. Methodologically similar work to the present one has also not been observed to date. There is no doubt that both the pandemic and the war in Ukraine have a significant impact on the health of Poles and their effectiveness as workers, posing an important scientific, clinical, economic and social problem. We believe that the inclusion of computational models here will make the following contribution to this article will:

- Improve the objectivity of the test results;
- Reduce measurement uncertainty;
- Enable a more accurate estimation of the current state of quality of life;
- Make it possible to predict future values of quality of life;
- Make it possible to identify a trend to reverse the direction of unfavorable changes;
- Make it possible to build a family of solutions based on similar computational mechanisms.

The aim of the study is twofold: (1) To develop a fuzzy model reflecting the changes in life satisfaction scores under the influence of the COVID-19 pandemic and the war in the neighboring country; (2) To develop more detailed guidelines than the existing ones for further similar research on more advanced computational models. We are concerned with developing practical solutions with higher scientific and clinical utility for both small data sets and big data.

## 2. Material and Methods

### 2.1. Materials

Two groups of Poles affected by burnout to varying degrees were assessed three times for life satisfaction; before and during the pandemic and during the war in Ukraine. They were the study group (physiotherapists, n = 20) and the reference group (IT specialists, n = 20). The clinical summary of the subjects is presented in Table 1.

This Polish group of professionals was taken as an example for further research and analysis of other groups. The recruitment process employed was that of a convenience sample. The criteria for inclusion were the following: age of at least 18 years and uninterrupted history of work as a physiotherapist (study group) or informatician (reference group). The criteria for exclusion were the following: age under 18 years, breaks in employment and diagnosed severe illnesses, including psychical. The use of the Polish group adds to the value of our study because we have access to results collected in three periods (before COVID, after COVID, and during the war) where particularly characteristic influences on quality of life status could be observed.

**Figure 1:**
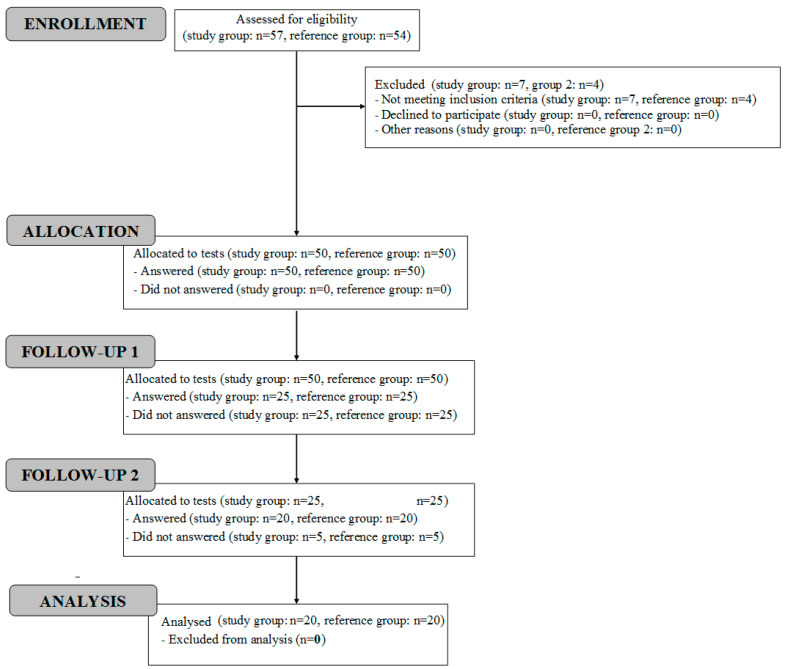
Participants’ flow diagram.

The participants’ flow diagram is presented in Figure 1.

### 2.2. Methods

The Perceived Stress Scale (PSS10), Maslach Burnout Scale (MBI), Life Satisfaction Scale (SWLS), and Nordic Musculoskeletal Questionnaire (NMQ), as well as statistical analyses and a fuzzy model, were used for the assessment. The characteristics of each scale relevant to the analysis and application in the fuzzy model are presented in Table 2.

Each participant was evaluated thrice: before the COVID-19 pandemic, in the second year of the pandemic, and during the war in Ukraine (in June/July 2022, i.e., after four months of the war).

### 2.3. Statistical Analysis

The results of tests, calculations and models were saved in an MS Excel spreadsheet. Statistical analysis was performed using the Statistica 13 program (StatSoft, Tulsa, OK, USA). The Shapiro-Wilk test was used to check the normality of the distribution of the tested data. To determine the statistical significance of the differences, the *p*-value was set at 0.05. This value is assimilated at this level in biomedical publications, which makes the results in this work comparable with others published in the same area and replicable. The analyzed values with a distribution close to the normal distribution were presented as mean values and standard deviation (SD). The analyzed values with distributions different to the normal distribution were presented employing the minimum value, the lower quartile (Q1), the median, the upper quartile (Q3) and the maximum value. The direction and strength of the correlation between the analyzed data were presented using the Rho Spearman coefficient.

### 2.4. Computational Methods

Proprietary software was used to carry out the computational analysis, which is part of the library for processing and computing ordered fuzzy numbers(OFNs) developed in 2012–2022.The verification of the computational correctness of the proprietary solutions used in this work was carried out by means of control calculations using (where possible) spreadsheets and reference values obtained from the Matlab + Fuzzy Logic Toolbox software.

Fuzzy models are increasingly used as an important part of computational modeling systems, including medical diagnostic purposes in healthcare [43,44]. Fuzzy logic is categorized as a method of artificial intelligence (AI) and, more broadly, computational intelligence (CI) [45]. It reduces the ambiguity and uncertainty (i.e., lack of ambiguous accuracy of linguistic description) associated with clinical decision-making based on diagnostic test results, medical imaging, patient history, interviews, etc. [46,47,48]. Descriptive information typical of humans (‘minor stress’, ‘moderate discomfort’) is transformed into numerical information that computational systems can process. This also increases the freedom of data handling and information exchange in the human–computer system relationship. The current study represents a further step towards the development of computational patient models (digital twins) to facilitate the prediction and diagnosis of psychological and psychological problems, including burnout. The proposed group of solutions will help improve the accuracy, efficiency and safety of such tools [49,50]. To model the evaluation process with multiple different data sources, a fuzzy system with multiple inputs and one output is required, giving the final grade as a single number. For the task, a fuzzy Mamdani-type system was proposed, because the assumptions, principles and interpretation of the scales used for the evaluation are described linguistically.

### 2.5. Algorithm of Data Processing

We checked in previous research [11,12,13,14,15] that the following configuration turned out to be effective:

- In the rules—operation of aggregation of premises—PROD;
- Implication operator—MIN;
- Operation of accumulation, also called aggregation of results, from the rules—MAX;
- Operation of defuzzification-center of gravity (COG).

For this reason, the above-mentioned parameters were also used in the model proposed in this article. We used flexible ones in the proposed fuzzy system, a tool for scaling the results as an interval [0; 1].

In this article, we mainly use standard trapezoidal fuzzy sets (fuzzy intervals). For their description, we will use a notation similar to the description of LR fuzzy sets [41] but change the order of the values.

Following that, a trapezoidal fuzzy set T may be described as:

T = (l, k_1_, k_2_, r)

where:

l, r–left (low) and right (maximum) boundaries of the support of T, k_1_, k_2_ represents the kernel interval of T. 

This description of the fuzzy set is consistent with the standard adopted in many popular scientific tools (Matlab, Octave, Scilab, etc.).

For the research in this paper, the six data inputs were separated. Three of them are:

- PSS10-Perceived Stress Score
  - Interval of values X_PSS_ = (0;40);
  - Basic linguistic interpretation: the lower values mean the better situation;
  - There is a suggestion of three potential output states in the specificity of the interpretation.
- SWLS
  - Interval of values XSWLS = (5;35);
  - Basic linguistic interpretation: the higher values mean a better situation;
  - In the specificity of the interpretation, there are suggestions of six potential output states, yet the numerical interval is quite narrow, so the context of the outputs was paired. Finally, three potential output states were defined.
- NMQ-Nordic Musculoskeletal Questionnaire
  - Range of values XNMQ = (0;40);
  - General interpretation: the lower value means the better situation;
  - The basics of the interpretation do not suggest any specific number of outputs.

Although the MBI is one questionnaire, it represents three completely different factors, so it is justified to treat them separately. Additionally, each of them has its own scale and interpretation:

- “Emotional exhaustion” Xem
  - Range of values Xem = (0;54);
  - General interpretation: the lower value means a better psychological condition.
- “Depersonalization” Xdep
  - Range of values Xdep = (0;30);
  - General interpretation: the lower value means a better psychological condition.
- “Lack of personal achievements” Xachiev
  - Range of values Xachiev = (0;48);
  - General interpretation: the higher value means a better psychological condition, the opposite direction to the other MBI factors.

All three factors share the specificity of three potential interpretations of the baseline.

All of the above inputs belong to three different concepts of quality of life assessment:

- PSS10 and SWLS–general opinion about the own life of the respondent.
- NMQ–physical state.
- MBI factors–job burnout.

**Figure 2:**
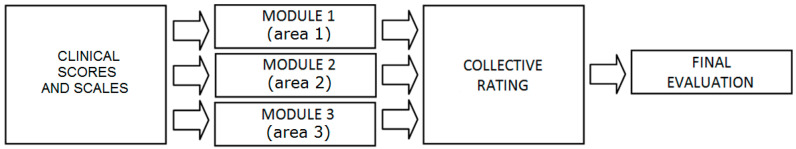
Structure of the used fuzzy system-modular structure of the hierarchy(own concept).

**Figure 3:**
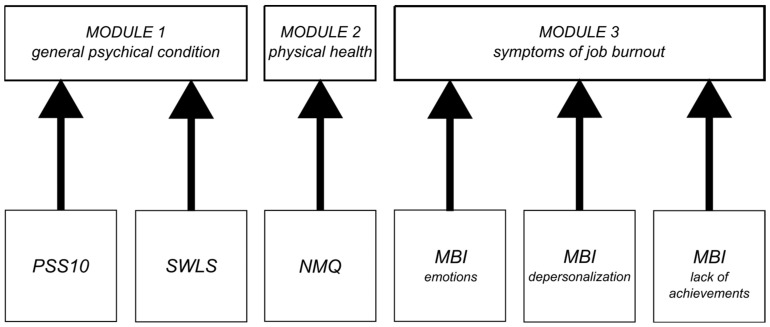
Structure of the used fuzzy system details (own concept).

In addition to reducing the number of rules, this concept allows for a modular structure (see Figure 2 and Figure 3). For a better understanding, we split the conceptual figure of the proposed fuzzy approach into two figures (Figure 2 and Figure 3).In the first one, we show the modular structure of the hierarchy (without details) conceptually, and in the second one, we show the details of the modules by omitting the top elements of the hierarchy. This makes the final evaluation dependent on three input sources of equal importance, as we expect from this evaluation.

The structure of the model is a three-level hierarchical system divided into three modules:

- Mental state assessment module-collecting data from PSS10 and SWLS;
- Physical condition assessment module that collects data from NMQ questionnaires;
- Burnout assessment module based on MBI, divided into three features: emotions, depersonalization, and lack of achievement; a simplified structure of the approach from the article by Prokopowicz and Mikołajewski [12].

The first tier is a simple fuzzy normalization of the input data for every individual feature available and significant in the PLUS evaluation. 

The input data *X* consists of six components:

*X = {X_PSS_, X_SWLS_, X_NMQ_X_em_, X_dep_, X_achiev_},*

where each of them is divided into fuzzy sets as follows (described as fours, a trapezoid shape):

*X_PSS_: X^L^_PSS_ = (0;0;0;14), X^M^_PSS_ = (0;14;26;40), X^H^_PSS_ = (26;40;40;40),*

*X_SWLS_: X^L^_SWLS_ = (5;5;5;15), X^M^_SWLS_ = (5;15;24;35), X^H^_SWLS_ = (24;35;35;35),*

*X_NMQ_: X^L^_PSS_ = (0;0;0;14), X^M^_PSS_ = (0;14;26;40), X^H^_PSS_ = (26;40;40;40),*

*X_em_: X^L^_em_ = (0, 0, 0, 16), X^M^_em_ = (0, 16, 27, 54), X^H^_em_ = (27, 54, 54, 54),*

*X_dep_: X^L^_dep_ = (0, 0, 0, 8), X^M^_dep_ = (0, 8, 14, 30), X^H^_dep_ = (14, 30, 30, 30),*

*X_achiev_: X^L^_achiev_ = (0, 0, 0, 31), X^M^_achiev_ = (0, 31, 39, 49), X^H^_achiev_ = (39, 49, 49, 49).*

In general, we divide all inputs into three fuzzy values: low, medium and high.

For the output, we use a variable *E* with three fuzzy values:

*E= {Y_1_, Y_2_, Y_3_}*

*Y_1_= (0, 0, 0, 0.5), Y_2_= (0, 0.5, 0.5, 1), Y_3_= (0.5, 1, 1, 1).*

To perform the defuzzification, the centre of gravity (COG) method is used. Therefore, it should be mentioned that for practical reasons, to avoid extra scaling in the computer processing, we use some extended fuzzy sets, *Y_1_ = (−0.5, 0, 0, 0.5)* and *Y_3_ = (0.5, 1, 1, 1.5)*, to make it possible to obtain exactly 0 and exactly 1 as defuzzi fied boundary values.

Where the input data interpretation should be ‘the higher, the better’ (*X_SWLS_, X_achiev_*), we use the rules:

*R_1_: IFxisX^L^THENe= Y_1_,*

*R_2_: IF x isX^M^THENe= Y_2_,*

*R_3_: IFxisX^H^THENe= Y_3,_*

For the rest (*X_PSS_, X_NMQ_, X_em_, X_dep_*), the rules are:

*R_1_: IFxisX^L^THENe= Y_3_,*

*R_2_: IF x isX^M^THENe= Y_2_,*

*R_3_: IFxisX^H^THENe= Y_1_,*

where *x* represents the input variable, and *e* represents the exit/result data.

The second tier is a three-module concept mentioned above. Here we have normalized input data with the meaning ‘the higher, the better’. This layer aggregates the input data with the same context of health evaluation.

The specific processing is dependent on the number of inputs. With a singular input source, as for Module 2, there is no real processing, as there is no aggregation needed. Such a result is transmitted directly to output from the module to the next level system. For modules with more inputs, these inputs are divided into two-valued variables. As an example, the Module 3 structure is presented:

Input data I (output from tier 1–MBI features results):

*I = {I_em_, I_dep_, I_achiev_},*

where each input consists of two fuzzy sets: *I^L^ = (0;0;0;1), I^H^ = (0;1;1;1)*.

The output variable *O = {O_1_, O_2_, O_3_, O_4_}*,consists of four fuzzy values:

*O_1_ = (0,0,0,0.333), O_2_ = (0,0.333,0.333,0.667), O_3_ = (0.333,0.667,0.667,1), O_4_ = (0.667,1,1,1).*

The rules aggregating three input data in Module 3 are defined as follows:

*R_1_: IF I_em_ is I^L^_em_ AND I_dep_ is I^L^_dep_ AND I_achiev_ is I^L^_achiev_ THEN o = O_1_,*

*R_2_: IF I_em_ is I^H^_em_ AND I_dep_ is I^L^_dep_ AND I_achiev_ is I^L^_achiev_ THEN o = O_2_,*

*R_3_: IF I_em_ is I^L^_em_ AND I_dep_ is I^H^_dep_ AND I_achiev_ is I^L^_achiev_ THEN o = O_2_,*

*R_4_: IF I_em_ is I^L^_em_ AND I_dep_ is I^L^_dep_ AND I_achiev_ is I^H^_achiev_ THEN o = O_2_,*

*R_5_: IF I_em_ is I^H^_em_ AND I_dep_ is I^H^_dep_ AND I_achiev_ is I^L^_achiev_ THEN o = O_3_,*

*R_6_: IF I_em_ is I^L^_em_ AND I_dep_ is I^H^_dep_ AND I_achiev_ is I^H^_achiev_ THEN o = O_3_,*

*R_7_: IF I_em_ is I^H^_em_ AND I_dep_ is I^L^_dep_ AND I_achiev_ is I^H^_achiev_ THEN o = O_3_,*

*R_8_: IF I_em_ is I^H^_em_ AND I_dep_ is I^H^_dep_ AND I_achiev_ is I^H^_achiev_ THEN o = O_4_*

The final-tier system’s purpose is the aggregation of the results from the modules. Such a structure (and configuration) is the result of evolution during research conducted in the last few years and described in [15,21,23]. It is worth noting that the structure of the final-tier fuzzy system is the same as for Module 3, presented above. It also aggregates three normalized inputs.

The basis for the implementation of data processing has been developed in previous publications [15,21], where various attempts to define the model were analyzed.

## 3. Results

### 3.1. General Results

It can be seen from these results that physiotherapists’ quality of life is declining, while IT professionals have improved after the COVID-19 period despite the war and galloping inflation. The results of the study are presented in the tables below (Table 3 and Table 4). The values expressed as median were significantly worse in the study group (physiotherapists) than in the reference group (IT specialists).

The differences between the two groups and correlations are presented in the tables during the fuzzy model discussion (Table 5 and Table 6).

The differences in correlations are significant; in group 1, statistically significant correlations were observed between MBI and PSS10 and MBI and SWLS scores, while in group 2, statistically significant correlations were observed between NMQ and MBI, NMQ and SWLS, NMQ and PSS10 scores. This may indicate that there are significant differences between the groups of physiotherapists and IT professionals in defining what well-being and job burnout mean to them and what factors influence their values.

This observation represents an important finding of our study. Physiotherapists work with sick people and are subject to considerable physical strain, whereas IT professionals work with machines and in project teams under high time pressure, and their physical strain is mainly on the spine and upper limbs (sedentary work at a monitor and keyboard).

An appropriately selected group of tests, as in our study, is able to capture the above-mentioned differences. These will influence both the strategies to combat burnout and the motivational systems, different in the above-mentioned occupational groups.

### 3.2. Fuzzy Evaluation Model

The proposed model develops a simplified fuzzy approach from the work of Prokopowicz and Mikołajewski [15,21,23]. It is a three-level hierarchical system based on the division into three main modules:

- Mental state assessment module, collecting data from PSS10 and SWLS;
- Burnout assessment module collecting data from MBI (in three areas: emotions, depersonalization and lack of achievement);
- A physical condition assessment module, collecting data from NMQ (Table 7).

**Figure 4:**
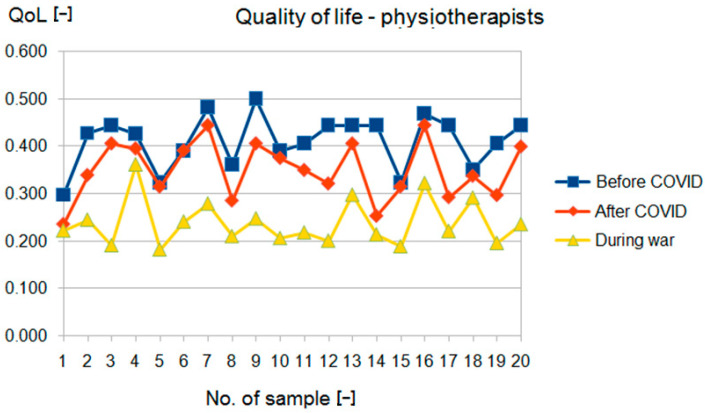
Fuzzy QoL for group 1.

**Figure 5:**
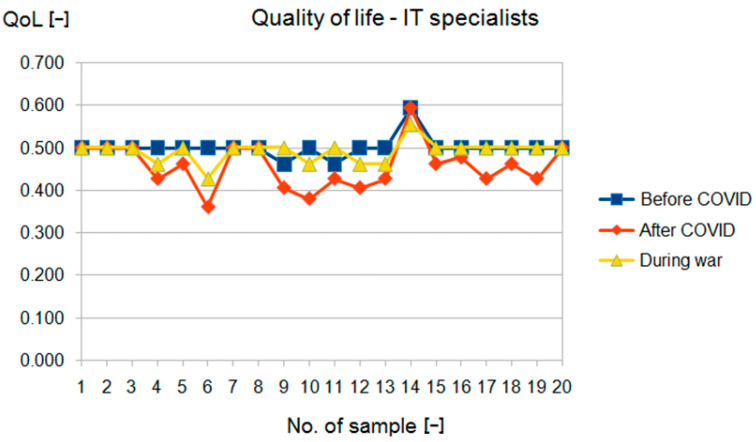
Fuzzy QoL for group 2.

**Figure 6:**
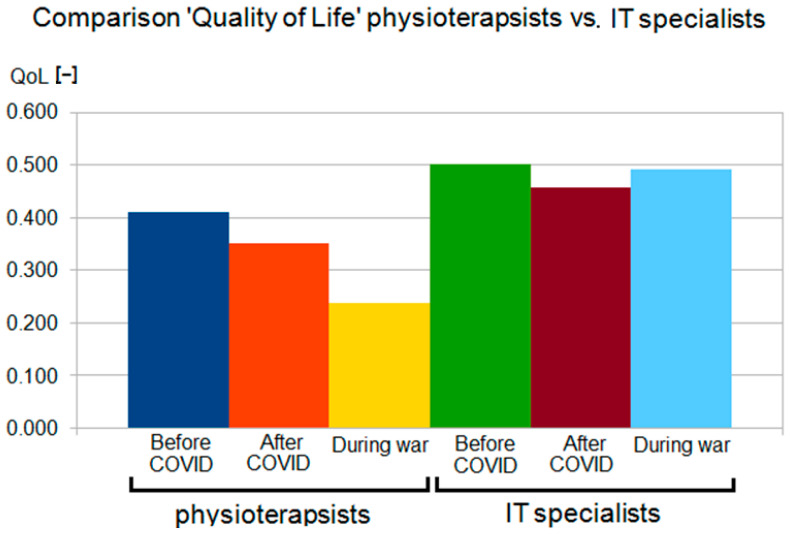
Fuzzy QoL in group 1 and group 2.

**Figure 7:**
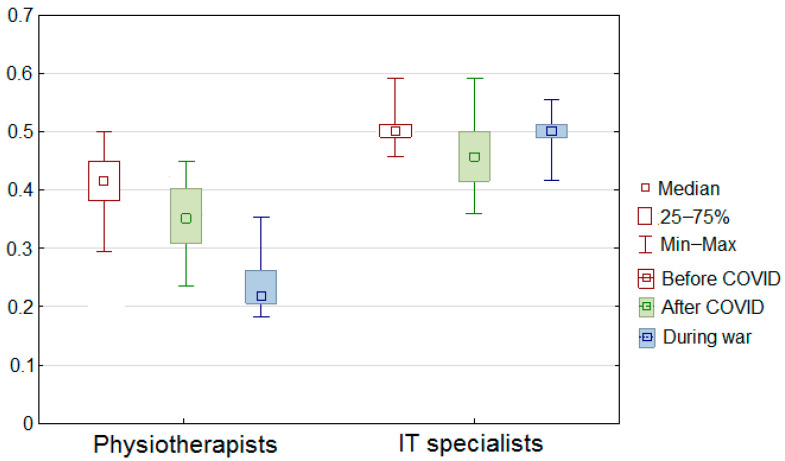
Fuzzy QoL in group 1 and group 2 (statistical view).

Detailed results are shown in Figure 4 and Figure 5, and the results are compared in Figure 6 and Figure 7. Changes in the study group (physiotherapists, Figure 4) were significantly greater than in the reference group (IT specialists, Figure 5), and the results in the study group were lower.

In addition, for the study group (physiotherapists), there was a clear downward trend in the results with successive examinations, whereas, for the reference group (IT specialists), such a trend was evident only in the second examination and with much less strength (Figure 6 and Figure 7).

The newly developed fuzzy model extracts new features as measurable properties of the observed phenomenon, reflecting the changes (Table 8).

It is noteworthy that the correlations between the changes in test scores and the changes reflected in the model are due to the differences in the internal correlations between groups 1 and 2, shown in Table 6 and Table 7. This may contribute to the development of more detailed models dedicated to specific occupational groups.

The advantage of the PLUS model is the high accuracy of translating the medical procedures (diagnostic testing) into computational evaluation algorithms, maintaining the assumptions of the linguistically described data processing model.

## 4. Discussion

Remote patient monitoring and assessment of the quality of life, self-management, daily physical activity, burnout and depression, and sleep, supported by health coaching, can be an important part of a preventive medicine system, reducing the need for acute care [51,52].

Change in quality of life often occurs as an accumulation of many factors, not only environmental (including the dynamics of change) but also individual (speed of learning and adaptation to changes in the environment, etc.) (Table 9). Optimal and objective assessment and deterioration detection of both the quality of life itself and optimal behaviors require sophisticated tools, including computational tools for analysis, classification and prediction [2]. In nature, the aforementioned behavioral optimization is implemented by coordinating multiple brain neural network systems, representing distinct computational algorithms or even sets of algorithms. Disruption of the above-mentioned processes represents various disease states (depression, occupational burnout, and other neurological and psychiatric conditions). Their mechanisms are often highly complex and distributed over time, and their causes may not be fully elucidated for the time being [2]. Despite the small number of publications, it is estimated that AI has great potential to improve the care of people with conditions that affect the quality of life of patients and their caregivers (e.g., Alzheimer’s disease).

### 4.1. Comparison with Other Studies

To date, there has been very little research in this area; only one study has been observed in the field of computational analysis on the impact of the pandemic (COVID-19) on life satisfaction (on the population of South Korea, using nomograms) [53]. The results included suggestions for the Korean government on what it should do at the national level to ensure a faster return to the citizens’ expected quality of life. We have not found a solution comparable to ours in the literature for scientific and clinical approaches.

There are no studies for comparison, and most of the results concerning the quality of life are descriptive and exploratory. They do not contain indications allowing one to distinguish between positive and mixed results, which in turn makes it difficult to compare the effectiveness of various therapeutic tools or models [3].The development of computational methods allows for the isolation and incorporation of relevant results to extend current biological and clinical quality-of-life measurements [54,55,56].No publications with the keywords “quality of life” + “war” + “artificial intelligence” were found in the reviewed bibliographic databases. The proposed PLUS model converts test results into a universal percentage scale (while maintaining their characteristics). The use of fuzzy logic provides easier analysis and inference when a given feature within the decision variant is described by several values(including a certain range of variability) or when the feature values are described ambiguously (linguistically). However, even the popular, fuzzy TOPSIS method (The Technique for Order of Preference by similar to Ideal Solution) [57,58] was not used for the ‘burnout’ diagnosis and treatment purposes.

The fuzzy character of job burnout was confirmed by Maija & Katri [59], and the fuzziness of well-being was confirmed by Dong & Yan [60]. This means that, although for a significant proportion of clinical decisions, a traditional deterministic approach may be sufficient, for both of the above-mentioned cases, a more complex approach is required, taking the linguistic nature of the description and multiple sources of uncertainty into account. The case we have described is particularly complex, for it considers not only the traditional factors of well-being and occupational stress but also an unprecedented accumulation of additional stressors: pandemics, war and economic crisis. This accounts for the uniqueness of our work.

We provided a summary of the methods, materials, and performance used in our previous articles on modeling job burnout [15,21]. There, we had three fuzzy systems that we compared in terms of accuracy in reflecting the results of aggregate quality of life and job burnout tests. A comparison of various approaches to QoL assessment is shown in Table 10.

In summary, the comparative analysis shows that:

- From a scientific point of view, our approach is not only technologically and cognitively new but also offers wider opportunities for development, opening up new research fields for computer science and computational neuroscience;
- From a practical (clinical) point of view, it is possible to screen faster and more widely for the changes in health associated with a faster pace of life and the emergence of problems on a global scale that can cause changes in mental health and health-related quality of life;
- From an economic point of view, the automation of early diagnosis may help to detect certain detrimental phenomena such as earlier burnout, implement prevention strategies, reduce absenteeism and improve work efficiency;
- From a societal point of view, it will enable the launch, in good time, of preventive and therapeutic actions at the level of entire communities, which may be necessary forsituations of massive, dynamic changes, such as a pandemic, an energy crisis, environmental pollution or the threat of war.

For most of us, these experiences are new, so any almost ready-to-use solution should be thoroughly tested and employed.

### 4.2. Limitations of the Own Study

The limitations of our study include the small size of the groups and the young age of the respondents. The data above will be expanded to include other groups in subsequent studies and other factors influencing their well-being.

Surveys underlying a particular module may not be popular or available in all regions of the world. Admittedly, thanks to modular design and standardization using fuzzy systems, we can change the source of data (type of survey, e.g., we replace a scale measuring physical health with another) while maintaining an unambiguous survey context. However, after such a change, the reliability of comparing results with those based on other surveys is reduced. At the same time, however, the advantage is that we can still collate these results to some extent, albeit with less reliability.

### 4.3. Directions for Further Research

Further research is needed to refine the guidelines for OSH supporting such complex life situations. The results of further research could be crucial in further projects related to computational fuzzy data analysis, clinical work and future research in medical and health sciences. It will allow knowledge and practice to be better adapted to the next possible crises, such as the refugee crisis on the border between Poland and Belarus, the energy crisis or the environmental crisis related to the pollution of the Oder river or the fires in France in 2022. Such phenomena, subjected to computational description, will allow the development of a whole family of globally useful models to help solve even difficult social problems.

As part of further research in the technical aspect, we plan to extend PLUS with inference and prediction with the use of artificial neural networks, as well as the study of non-uniformity (the rate of change) of individual research results with the aid of fractal analysis.

Systems incorporating artificial intelligence elements are gaining popularity and have proven their usefulness within computer-aided detection (CADe) and computer-aided diagnosis (CADx) systems. The hierarchical structure of the proposed fuzzy system provides a more flexible way of combining data from several different sources. This represents advantages for the proposed tool in terms of greater development possibilities and greater clinical utility, should the opportunity arise to expand the range of tests or analysis methods. This will provide both trend analysis and prediction of values over different periods. This will enable earlier implementation of strategies to prevent detrimental changes in workers’ health, which fits in with the rapid post-pandemic response strategies currently being developed.

The proposed tool aggregates the results obtained from many clinimetric tests into a single number. This allows for a clearer consideration of a greater number of aspects of the phenomenon under study. The final assessment will therefore be more accurate and complete.

An important area of further research on changes in PLUS is the use of the potential of ordered fuzzy numbers (OFNs) [9,54,55,56]. This will allow for modeling information on the direction and dynamics of their changes, while maintaining the intuitiveness of fuzzy models. It will also allow further analysis and processing of the results as an OFN. The OFN-based model will also provide flexibility, i.e., it will be easy to adapt to the next set of base coefficients. This means it is possible to enter further data from surveys covering the same module, and we can easily include them in the algorithm. This will be done without significantly changing the underlying measurement structure.

PLUS monitoring is a tool that, when used appropriately, offers the possibility of early detection of depressive states and behaviors or job burnout, etc. [1,61], including those based on the recent rapid development of sensors and their networks within the Internet of Things (IoT) [62].

## 5. Conclusions

Remote patient monitoring has the potential to reduce the use of acute care, but the effectiveness varies between populations and conditions, requiring more research and further analysis and technological developments in monitoring the quality of life of patients and healthy people.

The reality presents us with more and more difficult challenges to analyze and predict;hence we must have appropriate, proven, accurate and reliable tools for this purpose. The development of such computational tools is crucial for detecting, assessing and monitoring changes in the well-being of individual citizens and their communities, as it cannot be done manually on a massive scale. It is part of both preventive medicine (including occupational medicine) and personalized therapy. It is favored by the proliferation of smartphones, wearables and the Internet of Things, and advanced eHealth and Clinic 4.0 systems supporting medical specialists in collecting data and making clinical decisions. Additionally, the massive collection of large data sets will provide the basis for the acquisition of new knowledge about new or detailed biological mechanisms, which is difficult to extract using methods other than computational. In conclusion, we can state that the author’s fuzzy model (PLUS) represents a new tool that, when developed, can creatively support diagnosticians and clinicians (second opinion system). This is realized through an automatic computational analysis of life satisfaction based on artificial intelligence. In particular, it addresses the impact of occupational stress and burnout in both healthy individuals and diagnosed patients. For the above reasons, it may be an element of a new screening system for the psychological condition of society, alerting us to the emergence of a harmful trend, even with early symptoms. This can speed up the response and improve the effectiveness of cheaper prophylaxis instead of traditional treatment of patients. The limited financial resources of the healthcare system can be better distributed and directed to the more severe cases that are most in need.

Adaptation of the PLUS model to individual features is facilitated by the legibility and flexibility of language rules. They also allow for the construction of more complex models on this basis, including those dedicated to other groups of diseases.

## Figures and Tables

**Table 1:** Clinical summary of the subjects (at the beginning of the study).

	Study Group	Reference Group
	(*n* = 20, 100%)	(*n* = 20, 100%)
Age [years]		
Mean	27.40	26.55
SD	3.89	4.06
Min	22	22
Q1	24	23.5
Median	24	25.5
Q3	29.5	28
Max	34	35
Seniority [years]		
Mean	3.45	3.6
SD	2.61	2.52
Min	1	1
Q1	1	2
Median	3	3
Q3	5.5	4.5
Max	8	9
Gender:		
Females (F)	8 (40%)	9 (45%)
Males (M)	12 (60%)	11 (55%)

**Table 2:** Characteristics of scales used in the study.

Scale Name	Change Direction	Test Scoring
PSS10	a higher score means higher stress	1–4: low, 5–6 moderate, 7–10: high
MBI	a higher score means higher stress	Three subscales are measured separately: (1) emotional exhaustion (9 items), (2) depersonalization (5 items), (3) personal achievements (8 items)
SWLS	a higher score means a higher quality of life	whole range is 5–35, where 5–9 extremely dissatisfied with life, 20 neutral, 31–35 extremely satisfied with life
NMQ	a higher score means a higher number of pain problems	how often problems with locomotion are observed

**Table 3:** Results for group 1.

Scale	PSS10	MBI	SWLS	NMQ
		**Before COVID**		
Mean	29.20	48.75	16.3	0.70
SD	2.71	15.50	3.57	0.73
Min	25	32	12	0
Q1	28	37.5	14.25	0
Median	28	45.5	25.5	1
Q3	31.25	53.75	17	1
Max	34	79	25	2
Distribution	not normal	not normal	not normal	not normal
		**After COVID**		
Mean	30.85	56.75	14.90	0.70
SD	2.25	12.67	3.42	0.73
Min	27	40	11	0
Q1	30	44.25	13	0
Median	30.5	54.5	14	1
Q3	32.25	65.25	15.25	1
Max	35	79	25	2
Distribution	Normal	not normal	not normal	not normal
**During War in the Neighboring Country**				
Mean	33.2	63.50	10.95	0.55
SD	2.19	8.99	2.26	0.61
Min	30	50	8	0
Q1	32	55	10	0
Median	33	62.5	10	0.5
Q3	35	69.25	13	1
Max	37	78	17	2
Distribution	Normal	normal	not normal	not normal

**Table 4:** Results for group 2.

Scale	PSS10	MBI	SWLS	NMQ
**Before COVID**				
Mean	18.55	17.25	53.55	0.45
SD	3.50	2.94	17.55	0.51
Min	10	14	25	0
Q1	16	15	42.5	0
Median	19.5	16.5	56.5	0
Q3	20.25	18.5	69	1
Max	24	24	77	1
Distribution	Normal	not normal	not normal	not normal
**After COVID**				
Mean	16.1	13.95	62.5	0.50
SD	2.75	2.42	16.39	0.51
Min	10	10	41	0
Q1	15	12.5	49.5	0
Median	16	13.5	60	0.5
Q3	18	15	76.75	1
Max	21	20	87	1
Distribution	Normal	not normal	not normal	not normal
**During War in the Neighboring Country**				
Mean	18.05	16.10	55.95	0.50
SD	3.53	2.10	14.06	0.51
Min	11	13	36	0
Q1	16	14	42.5	0
Median	17,5	16	52	0.5
Q3	20	18	69.5	1
Max	24	20	79	1
Distribution	Normal	normal	normal	not normal

**Table 5:** Correlations between test results for group 1.

Before COVID				
Scale	PSS10	MBI	SWLS	NMQ
PSS10	-	0.480 *p* = 0.032	n.s.	n.s.
MBI	0.480 *p* = 0.032	-	n.s	n.s.
SWLS	n.s.	n.s.	-	n.s.
NMQ	n.s.	n.s.	n.s.	-
**After COVID**				
PSS10	-	0.563 *p =* 0.009	n.s	n.s.
MBI	0.563 *p* = 0.009	-	−0.437 *p =* 0.044	n.s.
SWLS	n.s.	−0.437 *p =* 0.044	-	n.s.
NMQ	n.s.	n.s.	n.s.	-
**During War in the Neighboring Country**				
PSS10	-	n.s	0.462 *p =* 0.040	n.s.
MBI	n.s.	-	n.s.	n.s.
SWLS	0.462 *p =* 0.040	n.s.	-	n.s.
NMQ	n.s.	n.s.	n.s.	-

n.s. = not significant.

**Table 6:** Correlations between test results for group 2.

Before COVID				
Scale	PSS10	MBI	SWLS	NMQ
PSS10	-	n.s.	n.s.	0.264 *p =* 0.026
MBI	n.s.	-	n.s	−0.257 *p =* 0.027
SWLS	n.s.	n.s.	-	0.811 *p =* 0.000
NMQ	0.264 *p =* 0.026	−0.257 *p =* 0.027	0.811 *p =* 0.000	-
**After COVID**				
PSS10	-	n.s.	n.s	n.s.
MBI	n.s.	-	n.s.	n.s.
SWLS	n.s.	n.s.	-	0.590 *p =* 0.006
NMQ	n.s.	n.s.	0.590 *p =* 0.006	-
**During War in the Neighboring Country**				
PSS10	-	n.s	n.s.	0.297 *p = 0*.020
MBI	n.s.	-	n.s.	n.s.
SWLS	n.s.	n.s.	-	0.792 *p =* 0.000
NMQ	0.297 *p =* 0.020	n.s	0.792 *p =* 0.000	-

n.s. = not significant.

**Table 7:** Fuzzy model outcomes for both groups: group 1 (physical therapists) and group 2 (informaticians).

No.	Physical Therapists			Informaticians		
	Before COVID	After COVID	During War	Before COVID	After COVID	During War
1	0.297	0.236	0.222	0.500	0.500	0.500
2	0.428	0.339	0.244	0.500	0.500	0.500
3	0.445	0.406	0.191	0.500	0.500	0.500
4	0.426	0.395	0.361	0.500	0.428	0.462
5	0.324	0.315	0.182	0.500	0.462	0.500
6	0.391	0.391	0.241	0.500	0.361	0.428
7	0.482	0.445	0.278	0.500	0.500	0.500
8	0.361	0.286	0.210	0.500	0.500	0.500
9	0.500	0.406	0.247	0.462	0.406	0.500
10	0.391	0.376	0.206	0.500	0.380	0.462
11	0.406	0.350	0.217	0.462	0.428	0.500
12	0.445	0.322	0.200	0.500	0.406	0.462
13	0.445	0.406	0.297	0.500	0.428	0.462
14	0.445	0.253	0.213	0.594	0.594	0.555
15	0.324	0.315	0.188	0.500	0.462	0.500
16	0.468	0.445	0.322	0.500	0.477	0.500
17	0.445	0.293	0.221	0.500	0.428	0.500
18	0.352	0.338	0.291	0.500	0.462	0.500
19	0.406	0.297	0.195	0.500	0.428	0.500
20	0.445	0.400	0.235	0.500	0.500	0.500
Summary of the data set						
Sum	8.223	7.014	4.761	10.017	9.149	9.831
Min	0.297	0.236	0.182	0.462	0.361	0.428
Q1	0.383	0.311	0.204	0.500	0.428	0.491
Median	0.428	0.350	0.222	0.500	0.462	0.500
Q3	0.445	0.401	0.255	0.500	0.500	0.500
Max	0.500	0.445	0.361	0.594	0.594	0.555
Mean	0.411	0.351	0.238	0.501	0.457	0.492
SD	0.056	0.061	0.049	0.025	0.054	0.026

**Table 8:** Correlations between model outcomes and change of test outcomes for model verification purposes.

Group 1 (Physical Therapists)	
Change of PSS10	−0.658 *p* = 0.023
Change of MBI	n.s.
Change of SWLS	0.438 *p* = 0.011
Change of NMQ	n.s.
**Group 2 (Informaticians)**	
Change of PSS10	n.s.
Change of MBI	n.s.
Change of SWLS	−0.521 *p* = 0.031
Change of NMQ	−0.550 *p* = 0.035

**Table 9:** General results in the study (only statistically significant changes included).

Scale	PSS10	MBI	SWLS	NMQ
Direction of change in group 1 (physiotherapists)	High erstress	Higher stress	Lower quality of living	Higher number of problems
Direction of change in group 2 (informaticians)	Low erstress	Lower stress	Higher quality of living	No change

**Table 10:** Comparison of various approaches to QoL assessment.

	Area	Approach		
		Work Burnout/Life Burnout	QoL	PLUS
Every day activity	Physical health	Not considered	Partially considered	Considered–module 2
	Job satisfaction	Considered	Partially considered	Considered–module 3
	Life satisfaction	Partially considered	Partially considered	Considered–module 1
	Specific context influence	Not applicable	Not applicable	Applicable through modular construction–by adding another module with a specific evaluation

## Data Availability

Not applicable.
